# Concussion and the Sleeping Brain

**DOI:** 10.1186/s40798-024-00736-2

**Published:** 2024-06-09

**Authors:** Catherine C. Donahue, Jacob E. Resch

**Affiliations:** 1 Department of Orthopedics, University of Colorado School of Medicine, Children’s Hospital Colorado, 13123 E. 16th Ave, Box 060, 80045 Aurora, CO USA; 2 Department of Kinesiology, University of Virginia, 550 Brandon Ave, Charlottesville, VA 22908 USA

**Keywords:** Sleep, Concussion, Physiology

## Abstract

**Background:**

Emerging research has suggested sleep to be a modifier of the trajectory of concussion recovery in adolescent and adult populations. Despite the growing recognition of the relationship between sleep and concussion, the mechanisms and physiological processes governing this association have yet to be established.

**Main Body:**

Following a concussion, a pathophysiologic cascade of events occurs, characterized by numerous factors including microglia activation, ionic imbalance, and release of excitatory neurotransmitters. Importantly, each of these factors plays a role in the regulation of the sleep-wake cycle. Therefore, dysregulation of sleep following injury may be a function of the diffuse disruption of cerebral functioning in the wake of both axonal damage and secondary physiological events. As the onset of sleep-related symptoms is highly variable following a concussion, clinicians should be aware of when and how these symptoms present. Post-injury changes in sleep have been reported in the acute, sub-acute, and chronic phases of recovery and can prolong symptom resolution, affect neurocognitive performance, and influence mood state. Though these changes support sleep as a modifier of recovery, limited guidance exists for clinicians or their patients in the management of sleep after concussion. This may be attributed to the fact that research has correlated sleep with concussion recovery but has failed to explain why the correlation exists. Sleep is a complex, multifactorial process and the changes seen in sleep that are seen following concussion are the result of interactions amongst numerous processes that regulate the sleep-wake cycle.

**Short Conclusion:**

The assessment and management of sleep by identifying and considering the biological, sociological, and psychological interactions of this multifactorial process will allow for clinicians to address the dynamic nature of changes in sleep following concussion.

## Key Points

Sleep is considered a modifier of recovery from concussion, yet little attention has been given to the overlapping physiologies between sleep and concussion, thereby limiting understanding as to why it effects recovery.

Our theoretical model proposes that the neurometabolic cascade modulates sleep-wake physiology across multiple levels via alterations in neurotransmitter release, glymphatic clearance, and extracellular ionic concentration.

## Background

Sleep is an essential component of recovery following a concussion [1]. Of the multitude of symptoms that an individual may experience following the injury, sleep symptoms such as drowsiness, difficulty falling or staying asleep, or changes in the quantity and/or quality of sleep are endorsed by up to 70% of patients [2]. For this reason, sleep disturbances have been suggested as modifiers of recovery following a diagnosed concussion in young and adult athletes [3–8]. Sleep-related research has demonstrated that individuals who experience sleep-related symptoms take a greater number of days to report symptom resolution at rest and ultimately return to sport (RTS) following a diagnosed concussion [5, 7, 9, 10]. However, the physiological mechanism(s) of why sleep disturbances occur and how they influence recovery remain poorly understood.

Though sleep symptoms are commonly reported following a concussion, the pattern and time frame in which they present may vary between individuals and play a role in recovery from the injury. The temporal nature of sleep-symptom presentation necessitates the importance of health care providers understanding the physiological link between sleep and concussion, how to identify and evaluate sleep disturbances, and the management of injured athletes who express one or more sleep symptom(s) to facilitate their recovery. This review will address: what is known about the physiological link between sleep and concussion, common measures of sleep, sleep throughout recovery from a concussion, and areas for future research.

## Main Text

### Parallels between Concussion and Sleep Physiology

#### Sleep

Sleep is regulated by the interaction of two separate biological mechanisms: the homeostatic need for sleep (Process S), and the circadian timing of when sleep happens (Process C) [11–13]. The interaction between Process S and Process C is referred to as the 2-Process Model [11, 12]. The homeostatic component, Process S, can be described as an individual’s “need to sleep” during times of sustained wakefulness [11–13]. During these periods of sustained wakefulness, glycogen stores are being depleted while the amino acid adenosine is accumulating; the longer an individual is awake, the more adenosine accumulates [11–13]. Increased levels of adenosine subsequently inhibit various regions of the brain responsible for wakefulness which then results in the transition from wakefulness to sleep. In short, as an individual stays awake over an extended period of time, the homeostatic drive for sleep increases.

The circadian component, Process C, is described as an “internal clock”, which sets the time at which sleep and wakefulness occur in the 24 h cycle [11–13]. This internal clock associated with Process C is in the suprachiasmatic nucleus (SCN) located in the anterior hypothalamus. Process C is largely influenced by light input from the surrounding environment (i.e., sun light), thus activating wake-promoting regions of the brain [12, 13]. As light dissipates, Process C influences the pineal gland to release the sleep-promoting neuropeptide known as melatonin, thereby inhibiting wake-promoting regions of the brain which then facilitates Process S [12, 13]. 

As established, the 2-Process Model regulates sleep and wakefulness. This regulation of sleep and wakefulness is reflective of the inhibition or activation of various neurotransmitters which excite or inhibit the processes of wake-promoting neurons [11]. The excitement of sleep-promoting neurons or inhibition of wake-promoting neurons may prompt sleep [14]. Conversely, waking can be promoted through the excitement of wake-promoting neurons, the inhibition of sleep-promoting neurons, or both. The excitation or inhibition of these neurons results in the release of specific neurotransmitters. The primary neurotransmitters involved with sleep and wakefulness include: gamma-aminobutyric acid (GABA), hypocretin, acetylcholine, dopamine, noradrenaline, serotonin, adenosine, and histamine [14]. This excitation or inhibition of monoaminergic neurons and subsequent release of neurotransmitters is initiated and maintained by the ascending reticular activating system (ARAS) which is located in the brain stem [15]. During sleep, the ARAS is inhibited, thus allowing the excitation of the sleep-promoting neurotransmitters (e.g., GABA) which act to inhibit regions of the brain that release wake-promoting neurotransmitters. Waking occurs when the inhibition of the ARAS is weakened, allowing it to initiate a widespread network of signals to wake-promoting regions of the brain, resulting in the excitation of wake-promoting neurotransmitters including hypocretin, acetylcholine, dopamine, noradrenaline, serotonin, and histamine. This excitation transitions the body from a state of sleep to a state of waking [14, 15]. 

Importantly, this activation or inhibition of the ARAS is dependent on the extracellular ionic concentrations of calcium, magnesium, and potassium in the brain [16]. When the ARAS is inhibited (i.e., during sleep), the extracellular concentrations of calcium and magnesium are elevated, while potassium levels are low. Conversely, during ARAS activation (i.e., wakefulness), the extracellular concentrations of calcium and magnesium are low, and potassium levels are high [16]. This complementary ionic balance from sleep to wakefulness, and vice versa, has been demonstrated in animal models. In rodent models, researchers have observed that the sleep-wake state can be manipulated by altering the concentrations of calcium, magnesium, and potassium, in the absence of neurotransmitters [16]. These findings suggest that the ionic interplay between calcium, magnesium, and potassium alone plays a crucial role in the sleep-wake cycle.

#### Concussion

A concussion is caused by any biomechanical force to the head, neck, or spine that results in neuronal strain in the brain [1, 17]. Neuronal strain of the axonal membrane results in an immediate physiological response known as the neurometabolic cascade [18, 19]. Immediately following a neuronal insult, there is a marked decrease in cerebral blood flow, as well as the binding of the excitatory neurotransmitter glutamate to N-methyl-D-aspartate (NMDA) receptors, creating a dysregulation of ionic levels [18, 19]. This ionic flux results in depolarization of neurons. To restore cellular homeostasis, voltage and ligand-activated channels are opened, causing the depletion of intracellular adenosine triphosphate (ATP), thus creating a hypermetabolic state [18, 19]. Moreover, there is an influx of calcium (Ca^2+^) and sodium (Na^+^) ions, and an efflux of potassium (K^+^) ions. This influx of Ca^2+^ and Na^+^ ions is due to increased glutamate activity that adversely affects cellular mitochondrial function and impairs the generation of ATP which is needed to restore ionic balance [18, 19]. This ionic imbalance and overall disruption of homeostasis typically lasts between 24 h up to four days after the concussive insult [19]. In addition to the neurometabolic cascade, an inflammatory response ensues in which microglia are activated and inflammatory cytokines are released to aid in tissue repair and removal of cellular debris [20].

#### A Tale of Two Physiologies

The relationship between sleep and concussion is complex and multifaceted, and requires a comprehensive approach that addresses the overlapping physiological pathways between the two. At the onset of sleep, activity of the hypothalamic-pituitary-adrenal (HPA) axis, an area within the brain that aids in the regulation of the sleep-wake cycle, is suppressed. However, the suppressed activity of the HPA axis may become disrupted as a result of the neuroinflammatory response to a concussion, resulting in potentially disrupted sleep. Immediately following a concussion, the pathophysiological process of the neurometabolic cascade triggers an increased activation of the HPA axis and the sympathetic nervous system (SNS) [21]. This activation results in an increased release of the neurotransmitter noradrenaline (NA), which stimulates arousal [14, 21]. Relatively low levels of NA can exert neuroprotective properties; however in higher levels such as those seen in an inflammatory response (e.g., neurometabolic cascade), NA can be disruptive, particularly to stages of a sleep cycle [22]. A sleep cycle consists of three stages of non rapid eye movement (NREM) and one stage of rapid eye movement (REM) [11]. Individuals will typically experience four to five sleep cycles each night, with time spent in each stage of sleep progressively changing [11]. The stage of sleep known as rapid eye movement (REM) is specifically crucial to maintaining low levels of NA as it allows for optimum immune response. During REM sleep neurotransmitters such as GABA and acetylcholine act as anti-inflammatory regulators in the sense that they have been shown to block nuclear factor-KB (NF-KB) pathways, thereby contributing to the maintenance of the inflammatory response during sleep [23]. However, if NA levels are too high, this inflammatory regulation is affected [24]. In this way, both acute and chronic inflammation, such as that seen following concussion, may be modulated by NA, which in turn is modulated by sleep.

The mediating effects of NA can further be appreciated through its interactions with microglia. Microglia are immune cells within the central nervous system (i.e., brain and spinal cord) that perform immune surveillance and macrophage activities including the production of cytokines and/or chemokines [20, 25]. When an inflammatory process is triggered, microglia move from a state of surveillance to a state of activation [25]. Microglia have two adrenergic receptors: β2 which is active in a resting state, and α2A which is active in an inflammatory state [26]. Interestingly, NA mediates both down and upregulation of cytokine production. In a resting state, NA binds to β2 receptors and downregulates the expression of pro-inflammatory cytokines, whereas in an inflammatory state when the α2A receptor is active, NA will bind and cause an upregulation of cytokine production [27]. As previously mentioned in the context of concussion, immediately following injury, there is activation of the HPA axis and the sympathetic nervous system, which results in an increased release of NA [21]. Due to the activation of microglia in response to the injury, NA now binds to the α2A receptor which promotes cytokine production and release. When increased activation of the central nervous system is chronic, lasting days to weeks to months, maladaptive sleep patterns occur, which are known as sleep disturbances [28]. These sleep disturbances can manifest in the timing and length of sleep stages such as increased duration of REM sleep and decreased or increased duration of slow wave sleep (non rapid eye movement [NREM], stage 3).

There are many cytokines that act on the sleep-wake cycle. These include: interleukins (IL) 1,2,4,6,8,10,13,15,18; tumor necrosis factor (TNF); growth hormone (GH), and adenosine [28, 29]. This provides strong evidence to suggest that cytokines are heavily involved in the regulation of sleep. Specifically, IL-1 and TNF have been found to be sleep regulating substances (SRS) as they fulfill the criteria of being able to enhance or inhibit sleep if injected, they act on sleep regulatory circuits, and their levels have been shown to be altered in a pathological state that is associated with increased sleep [30]. Partial night-time sleep deprivation has been found to activate inflammatory pathways involving nuclear factor-KB (NF-KB), a protein transcription factor that influences SRS. Specifically, the activation of NF-KB results in an upregulation of the cytokines IL-1 and TNF and has been found to occur in areas such as the lateral hypothalamus [30]. Additionally, an increase in IL-1 may stimulate increased release of NA, further disrupting sleep and the inflammatory process. The role of neurotransmitter, specifically that of NA can further be appreciated through its interaction with the glymphatic system.

**Fig. 1:**
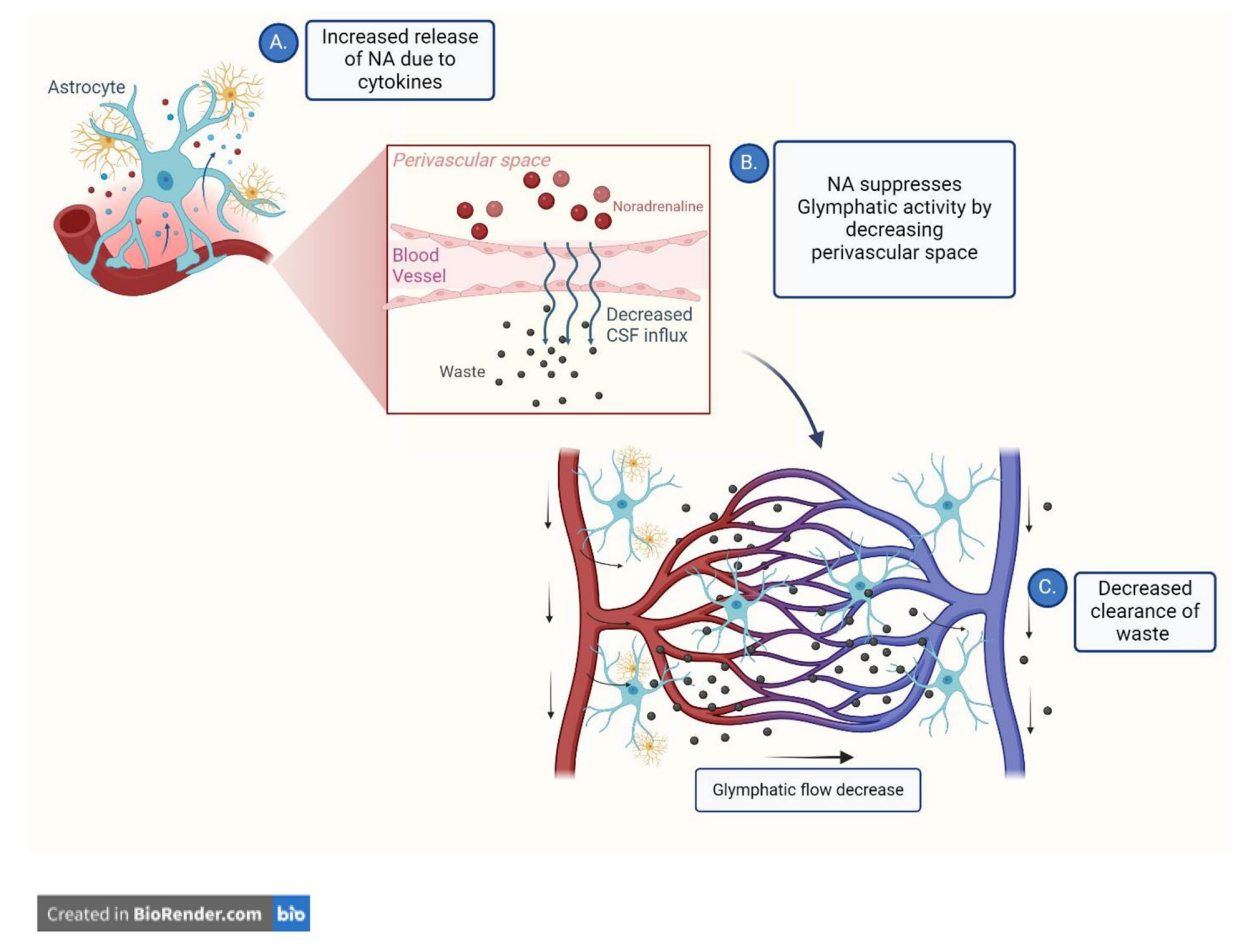
**A**. Increased release of noradrenaline (NA) due to inflammatory response (i.e. release of inflammatory cytokines) suppresses glymphatic clearance by decreasing perivascular space. **B**. Decreased perivascular space causes a decrease in cerebrospinal fluid (CSF)/interstitial fluid (ISF) exchange. **C**. A decrease in glymphatic flow decreases clearance of metabolic waste associated with the pathophysiology of the injury

The glymphatic system is a series of perivascular channels throughout the brain that serve to clear away and expel waste in the central nervous system (CNS) [31]. The glymphatic system is most active during sleep, bringing an influx of cerebral spinal fluid (CSF) into the brain to clear away metabolic waste (e.g., amyloid beta) that has naturally accumulated while awake [31]. Disruption of glymphatic activity has been implicated in various pathologies, such as anxiety, depression, and moderate traumatic brain injury (TBI), in which deficient sleep was associated with decreased perivascular flow of CSF, indicating dysfunction in waste clearance [32–34]. The enhanced efficiency of the glymphatic system due to sleep may be intertwined with the actions of NA [31]. During wakefulness, the release of NA acts to suppress glymphatic activity by decreasing perivascular space. Conversely, interstitial space is increased when NA release is inhibited [31]. This poses the theory that increased levels of NA as a result of the inflammatory response to concussion may hinder the glymphatic system’s ability to expel the cellular debris created by the injury. (Fig. 1.)

**Fig. 2:**
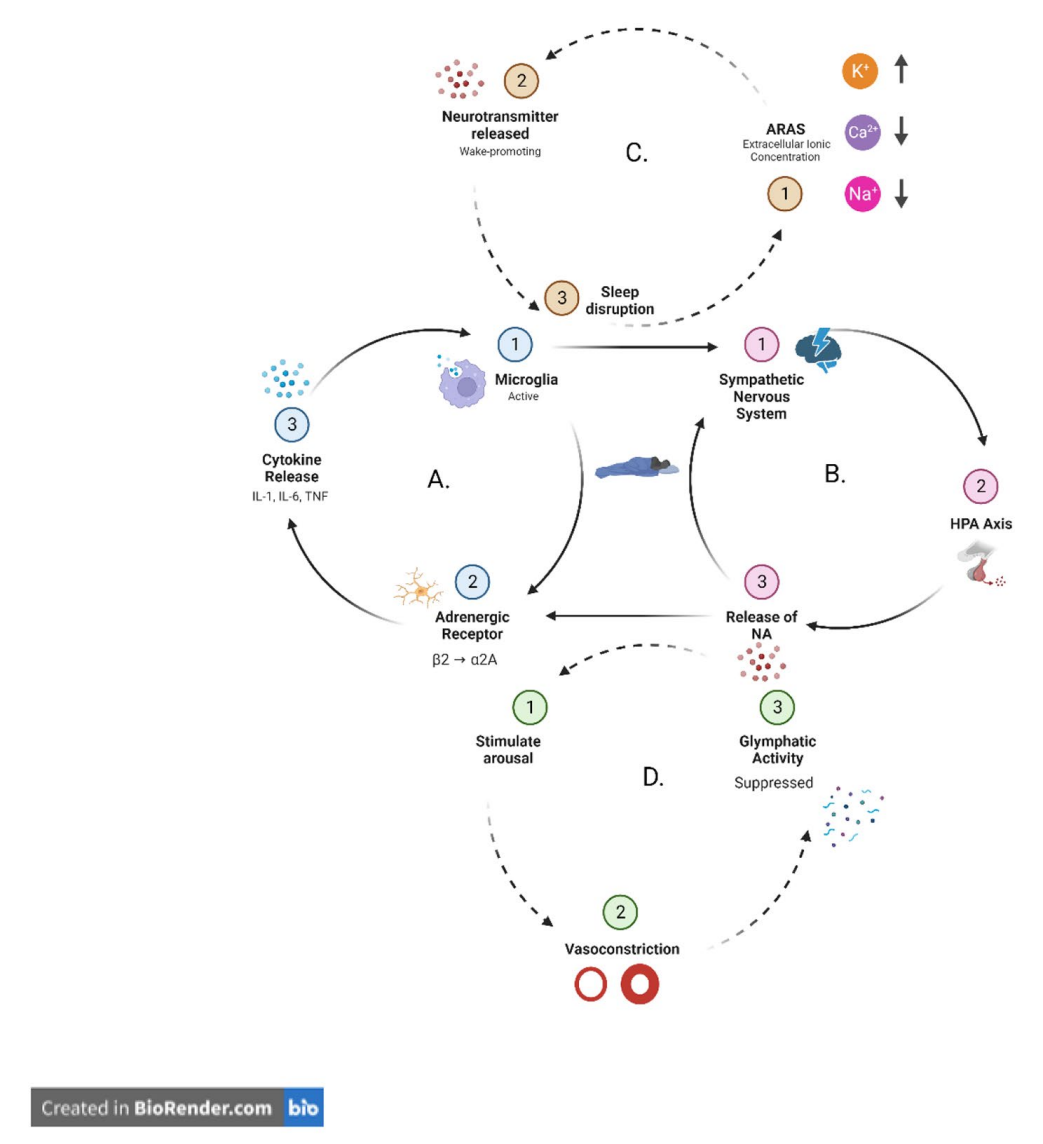
Feedback loop between the pathophysiology of concussion and sleep. **A**. Following a concussion, a neuroinflammatory response is initiated and microglia cells are activated (A1). The α2A adrenergic receptor of the microglia becomes active (A2), which subsequently creates an upregulation of pro-inflammatory cytokines (A3). **B**. The pathophysiological process of a concussion triggers an increased activation of the sympathetic nervous system (SNS) (B1) and hypothalamic-pituitary-adrenal (HPA) axis (B2), resulting in an increased release of noradrenaline (NA), which stimulates arousal (B3). **C**. The extracellular ionic concentration that occurs because of the neurometabolic cascade is high in K^+^ and low in Ca^2+^ and Na^+^ (C1). The same ionic concentration has been observed during the initiation of wakefulness by the ascending reticular activating system (ARAS), thus potentially further stimulating wake-promoting neurotransmitters (e.g., NA) (C2), and causing sleep disruptions (C3). **D**. The release of wake-promoting neurotransmitters, specifically NA (D1), suppresses glymphatic activity by stimulating vasoconstriction (D2), potentially hindering the glymphatic system’s ability to expel cellular debris created by the injury (D3)

Additional rationale for why sleep dysfunction following a concussion occurs may relate to the similarities of the ionic concentrations of the neurometabolic cascade and the sleep-wake cycle, ultimately affecting glymphatic function. The extracellular ionic concentration of the neurometabolic cascade is high in potassium (K^+^) and low in calcium (Ca^2+^). The same ionic concentration has been observed during the initiation and maintenance of wakefulness [16]. Theoretically, this may partially explain why sleep is disrupted following the injury. This theory may be further explained by the findings of Lundgaard and colleagues in which they observed the state of wakefulness or sleep is ionic dependent [16]. As such, it can be hypothesized that the resulting disruptions in sleep following a concussion may lead to decreased glymphatic clearance of the known metabolic waste associated with the pathophysiology of the injury. The multifaceted interaction of the physiology of sleep and concussion effectively creates a feedback loop (Fig. 2.) in which sleep disturbances manifest in young and adult populations.

### Measures of Sleep

As sleep is a multifaceted process, several clinical measures exist for evaluating its many constructs. Sleep constructs include, but are not limited to, total sleep duration, sleep staging, sleep efficiency, and sleep latency. Sleep can be measured using both subjective (e.g., questionnaires or diaries) and objective methods (e.g., actigraphy or lab assessment). However, as previously stated, no previously validated concussion-specific guidelines for the assessment or management of sleep currently exist, and as such the following section is intended to simply provide a brief overview.

#### Questionnaires and Diaries

Sleep diaries and questionnaires are designed to subjectively measure an individual’s sleep behaviors and patterns, and are the most cost-effective and simple way to assess sleep. Sleep diaries may vary in length and may include the self-reporting of bed and wake times, lights-out time, night time awakenings, or daytime naps [35].. Sleep diaries are best utilized to capture night-to-night variations in sleep, providing more precise information regarding changes in sleep [36]. Compared to a sleep questionnaire, diaries are particularly useful if they are used consistently for a period of time (i.e.-one to two weeks) as opposed to a singular time point which may not be an accurate depiction of sleep patterns [37]. Sleep questionnaires are often used as the first step in an initial assessment of sleep due to their time and cost-effectiveness. Questionnaires can serve as screening tools for further evaluation or for clinical diagnosis of sleep impairment and measure a number of aspects of sleep quality such as insomnia, sleep patterns, sleep apnea, or increased drowsiness [37]. Importantly, sleep questionnaires serve to quantitatively capture an individual’s perception of their sleep over a given period of time, ranging from one week to one month, making the results highly dependent on the individual’s memory of their sleep over a period of time. Though numerous sleep questionnaires exist, only the Pittsburgh Sleep Quality Index and Epworth Sleepiness Scale have been validated in traumatic brain injury populations [38, 39]. However, even the Pittsburgh Sleep Quality Index and Epworth Sleepiness Scale data have limitations: (1) neither had pre-injury sleep data to compare post-injury findings with; and (2) the average time since injury ranged from 3 to 38 months [38, 39]. To our knowledge the Sleep and Concussion Questionnaire is the only concussion-specific sleep questionnaire exists, and does demonstrate evidence of convergent validity with objective and subjective measures of sleep [40]. However, the Sleep and Concussion Questionnaire has similar limitations regarding length of time since injury (3–24 months) and lack of pre-injury sleep data for comparison.

#### Actigraphy

Actigraphy measures sleep by recording physical activity (or lack thereof) through the use of an accelerometer [41]. The recorded accelerometer data are then processed to determine an individual’s sleep or waking states [42]. Actigraphy devices can be worn on the wrist, hip, or finger, and allow for the continuous monitoring of sleep over multiple nights outside a laboratory setting. Examples of actigraphy devices include FitBit™ (San Francisco, CA), Garmin™ (Olathe, KS) watch, and the Oura™ ring (Oulu, Finland). The majority of consumer-available actigraphy devices used in measuring sleep began as fitness trackers. Consumer-available actigraphy devices offer limited utility in terms of which sleep metrics are recorded. The sleep metrics are limited to the binary classification of sleep or awake, sleep efficiency, latency, and wake after sleep onset [42]. Some devices were developed for the purpose of measuring sleep and offer additional sleep metrics (e.g., sleep staging). Generally speaking, when compared to polysomnography (PSG), actigraphy tends to overestimate sleep and underestimate wakefulness [42]. Clinically, for enough adequate information regarding an individual’s sleep, it is recommended that an actigraphy device be worn for one to two weeks, as this time frame captures week days and weekends and can inform clinical decision making [43]. Actigraphy has been used in several studies that assessed sleep after concussion in adults and adolescents. Findings demonstrate those with a concussion had poor sleep efficiency, greater sleep latency, frequent wakenings, as well as greater night to night variability [9, 44–46].

#### Polysomnography

PSG is considered the gold standard for assessing sleep [47]. Performed in a laboratory setting, PSG measures a number of bodily functions including sleep staging, respiratory rate and effort, heart rate, limb movement, and oxygen saturation [47]. As the gold standard, PSG has time and cost barriers limiting accessibility and feasibility, and it is typically only used for assessment of sleep disorders in response to the outcomes of alternative sleep measures. Moreover, as PSG is a laboratory assessment it involves an individual sleeping in an environment (i.e.- the laboratory) that does not reflect their natural sleep environment, and as such may artificially cause disrupted sleep and influence results [47]. Studies that assessed sleep following concussion using PSG have typically involved individuals experiencing chronic (i.e. month to years) sleep disturbances following the injury. However, the findings are inconsistent, with some demonstrating lower sleep efficiency (time spent asleep compared to total time in bed), increased as well as decreased sleep latency (time taken to fall asleep) and frequent nighttime wakenings [8, 48–51], as compared to others who have found no differences [2, 49, 52, 53]. and have demonstrated inconsistent findings. Time from injury in the aforementioned studies varied greatly, ranging from weeks to years, which may explain the inconsistent findings.

### Natural History of Sleep and Concussion

Following a concussion, athletes may experience a myriad of symptoms, including but not limited to headache, dizziness, confusion, or sleep disturbances. Though approximately 80% of individuals will experience a resolution of these symptoms within 14 days, the remaining 20% may take longer to recover [1]. This has resulted in substantial research around which specific symptoms associate with a prolonged recovery from concussion. Sleep is a component of “rest”. Rest is advised immediately following a concussion (< 48 h); however, aside from no longer recommending frequent night waking, no evidence based recommendations currently exist for the assessment or management of sleep following injury [1]. Most recently, questions addressing changes in sleep have been incorporated into the Concussion in Sport Group consensus statement as part of the Sport Concussion Office Assessment Tool (SCOAT) [17]. The addition of these questions provides a first step in evaluating sleep following concussion; however the lack of clinical or patient-specific guidance with respect to next steps may hinder effective assessment and management. Additionally, the aforementioned questions included in the SCOAT are adapted from the Athlete Sleep Screening Questionnaire - a questionnaire designed to assess sleep in elite (uninjured) athletes - which has not yet been validated in a population of concussed individuals [54]. To fully appreciate the impact of sleep disturbances on the management of concussion, it should be considered across the spectrum from prior to the injury (baseline) to throughout recovery.

#### Baseline

Evidence based recommendations for the management of concussion include a multimodal approach consisting of neurocognitive, balance, and symptom assessments to assess for recovery and safe RTS following a diagnosed injury [1]. Baseline values provide a patient-specific reference point for what is “normal” for post-injury assessments. Given that a safe return to sport is predicated on this comparison between post-injury and baseline values, it is important for clinicians to understand potential modifiers of a baseline assessment performance which includes sleep. Computerized neurocognitive testing (CNT) is one clinical measure of the battery of tests used in the management of concussion. CNTs measure various cognitive constructs (e.g.- memory, processing speed) and most include a separate measure of concussion-related symptomology (e.g., sleep quality and quantity). In high school and collegiate athletes, it has been demonstrated that less than seven hours of sleep the night prior to CNT completion is associated with increased reports of cognitive, somatic, and sleep-related symptoms [55, 56]. Moreover, deficient sleep has been demonstrated to associate with increased reaction time and decreased visual memory and verbal memory outcome scores on ImPACT, a commonly used CNT by athletic trainers [55]. It is important for clinicians to stress the importance of sleep to their athletes prior to the baseline assessment to avoid the potential addition of test error as a result of poor sleep to their observed outcome scores.

#### Post-injury

The relationship between sleep and concussion has been implicated throughout the spectrum of injury and throughout recovery in varying samples [2, 3, 5, 7, 9, 10, 57–59]. Changes in sleep quality and/or quantity have been associated with greater concussion-related symptomology (i.e., symptom quantity and severity) and a longer time until symptom resolution. The onset of sleep disturbances following concussion is highly variable and may evolve throughout recovery from a concussion. As such, clinicians should be cognizant of changes in sleep following injury to better identify and manage athletes that may be at risk for prolonged recovery.

Following a concussion, up to 70% of individuals report changes in sleep quality and quantity [2]. Changes in sleep during the acute phase of injury include greater difficulties falling asleep, increased hours of sleep, and nightly variability of overall sleep quality throughout recovery from concussion [3, 44]. Reports of decreased quality of sleep and an increase in quantity, suggest individuals diagnosed with a concussion may demonstrate a greater need for sleep immediately following their injury. In one study of collegiate athletes in the first 48 h, it was found that those with a concussion demonstrated longer time to fall asleep and greater variability in the number of minutes awake after having initially fallen asleep, when compared to healthy controls [44]. The study by Hoffman et al. [44] is one of the only studies that have examined sleep in the acute phase of injury. As such, there remains a gap in the research regarding sleep in the acute phase of injury, particularly in collegiate athletes, with most research examining sleep in the sub-acute (i.e.-3-14 days) or chronic (14 + days) phase of injury.

In the sub-acute phase of injury, changes in sleep begin to become more prominent. Adolescents between 11 and 18 years of age have been demonstrated to have up to a fourfold increase in recovery time if subjective sleep symptoms (e.g., difficulty falling asleep or staying asleep) are reported when compared to those who did not report the same symptoms [57]. Similarly, poor sleep quality has also been reported to result in a higher number of post-concussion symptoms, greater generalized anxiety and poorer quality of life when re-examined three months after initial evaluation [7]. Moreover, when examining the relationship between self-reported sleep quantity and post-concussion symptoms in the first week following injury, an increase in cognitive (e.g., memory recall) and neuropsychological (e.g., anxiety, depression) symptoms has been observed with a reduced number of hours of sleep [5]. Through use of wearable devices (e.g., wristbands, smartwatches, rings), sleep quality and quantity as measured by actigraphy has demonstrated that individuals who are awake longer throughout the night or are less efficient at sleeping experience concussion-related symptoms for a greater time period following injury.

Continued sleep disturbances beyond the typical symptom resolution time following a concussion (i.e. >28 days) are common and present similarly to trends seen in the acute and sub-acute phases. The endorsement of sleep disturbances following a concussion is associated with a greater severity of concussion-related symptoms. Additionally, a greater number of hours of sleep (i.e., sleeping more than usual) has been associated with decreased neurocognitive performance [10]. Decreased sleep efficiency and increased time spent awake have been found to associate with poor sleep quality up to one year after injury [9]. Future research should address sleep behaviors of athletes prior to their injury to appreciate how premorbid sleep habits may also influence recovery from concussion.

In summary, evidence suggests sleep is an additional piece of the puzzle for recovery from concussion. Clinicians must recognize the importance of sleep prior to and following a concussion. The incorporation of clinical measures of sleep into a concussion management protocol may assist in the recognition of sleep disturbances and help to identify ways to improve sleep quality and quantity of athletes recovering from the injury. As research in terms of sleep and concussion is in its infancy, the following directions for research addressing this interaction include:

- Investigation of how the severity of sleep symptoms influences the severity of all other symptoms commonly experienced following concussion.
- Assessment of additional measures of sleep such as timing of sleep staging immediately following injury and throughout recovery.
- Examination of anatomical regions of the brain that are involved in the regulation of the sleep-wake cycle for evidence of neuroinflammation.

## Conclusion

Following a concussion, and throughout recovery, sleep disturbances are often reported [2, 3, 5–7, 57]. These disturbances include but are not limited to delayed onset of sleep, frequent nighttime wakening, daytime sleepiness, and insomnia [2, 5, 7, 9, 10, 57]. The current literature surrounding sleep and concussion provides important rationale for examining why sleep disturbances are present immediately following injury and may persist for years afterwards. Thus far, research has demonstrated that both subjective as well as objective measures of sleep disturbances are associated with prolonged recovery [2, 5–7, 10, 44, 57]. Currently, an evidence-based approach does not exist for the management of sleep-related problems following concussion, but a better understanding of the physiological contributions of sleep, as provided in this review, may provide important first steps in the evaluation and management of sleep in individuals with concussion.

## Data Availability

Not applicable.

## Abbreviations

ATP: Adenosine triphosphate
ARAS: Ascending reticular activating system
Ca^2+^: Calcium
CNS: Central nervous system
CSF: Cerebral spinal fluid
CNT: Computerized neurocognitive testing
GABA: Gamma-aminobutyric acid
GH: Growth hormone
HPA: Hypothalamic-pituitary-adrenal axis
IL: Interleukin
NMDA: N-methyl-D-aspartate
NREM: Non rapid eye movement
NA: Noradrenaline
NF-KB: Nuclear factor-KB
PSG: Polysomnography
K^+^: Potassium
REM: Rapid eye movement
RTS: Return to sport
Na^+^: Sodium
SCN: Suprachiasmatic nucleus
SNS: Sympathetic nervous system
TBI: Traumatic brain injury
TNF: Tumor necrosis factor
